# Unique Molecular Patterns Uncovered in Kawasaki Disease Patients with Elevated Serum Gamma Glutamyl Transferase Levels: Implications for Intravenous Immunoglobulin Responsiveness

**DOI:** 10.1371/journal.pone.0167434

**Published:** 2016-12-21

**Authors:** Yue Wang, Zhen Li, Guang Hu, Shiying Hao, Xiaohong Deng, Min Huang, Miao Ren, Xiyuan Jiang, John T. Kanegaye, Kee-Soo Ha, JungHwa Lee, Xiaofeng Li, Xuejun Jiang, Yunxian Yu, Adriana H. Tremoulet, Jane C. Burns, John C. Whitin, Andrew Y. Shin, Karl G. Sylvester, Doff B. McElhinney, Harvey J. Cohen, Xuefeng B. Ling, Pediatric Emergency Medicine Kawasaki Disease Research Group

**Affiliations:** 1 School of public health, school of medicine, Zhejiang University, Hangzhou, China; 2 Stanford University, Stanford, CA, United States of America; 3 School of Electrical Engineering, Southeast University, Nanjing, China; 4 Shanghai Children's Hospital, Shanghai Jiao Tong University, Shanghai, China; 5 University of California San Diego, La Jolla, CA, United States of America; 6 Rady Children’s Hospital San Diego, San Diego, CA, United States of America; 7 Department of Pediatrics, Korea University Medical Center, Guro Hospital, Seoul, Korea; 8 Department of Heart Center, Beijing Children’s Hospital, Beijing, China; 9 Memorial Sloan-Kettering Cancer Center, New York, NY, United States of America; Institut National de la Santeet de la Recherche Medicale (INSERM), FRANCE

## Abstract

**Background:**

Resistance to intravenous immunoglobulin (IVIG) occurs in 10–20% of patients with Kawasaki disease (KD). The risk of resistance is about two-fold higher in patients with elevated gamma glutamyl transferase (GGT) levels. We sought to understand the biological mechanisms underlying IVIG resistance in patients with elevated GGT levels.

**Method:**

We explored the association between elevated GGT levels and IVIG-resistance with a cohort of 686 KD patients (Cohort I). Gene expression data from 130 children with acute KD (Cohort II) were analyzed using the R square statistic and false discovery analysis to identify genes that were differentially represented in patients with elevated GGT levels with regard to IVIG responsiveness. Two additional KD cohorts (Cohort III and IV) were used to test the hypothesis that sialylation and GGT may be involved in IVIG resistance through neutrophil apoptosis.

**Results:**

Thirty-six genes were identified that significantly explained the variations of both GGT levels and IVIG responsiveness in KD patients. After Bonferroni correction, significant associations with IVIG resistance persisted for 12 out of 36 genes among patients with elevated GGT levels and none among patients with normal GGT levels. With the discovery of *ST6GALNAC3*, a sialyltransferase, as the most differentially expressed gene, we hypothesized that sialylation and GGT are involved in IVIG resistance through neutrophil apoptosis. We then confirmed that in Cohort III and IV there was significantly less reduction in neutrophil count in IVIG non-responders.

**Conclusions:**

Gene expression analyses combining molecular and clinical datasets support the hypotheses that: (1) neutrophil apoptosis induced by IVIG may be a mechanism of action of IVIG in KD; (2) changes in sialylation and GGT level in KD patients may contribute synergistically to IVIG resistance through blocking IVIG-induced neutrophil apoptosis. These findings have implications for understanding the mechanism of action in IVIG resistance, and possibly for development of novel therapeutics.

## Introduction

Kawasaki disease (KD) is an acute systemic vasculitis of infants and children, that occurs world-wide [1], but the biology of this condition is not well understood. Coronary artery aneurysms (CAA) occur in 25% of untreated patients, making KD the leading cause of acquired heart disease in children [2, 3]. Therapy for KD includes high-dose aspirin and intravenous immune globulin (IVIG) [4], which reduces the incidence of CAA to 5–7% when given within the first 10 days of illness [5, 6]. However, 10%-20% of patients are resistant to IVIG [7, 8], and are consequently at greater risk of developing CAA, and require additional adjunctive treatments [5, 9]. We [10–12] and others [13–15] have found that serum gamma-glutamyl transferase (GGT) levels are elevated in the acute phase of KD patients and that higher GGT levels are associated with IVIG resistance.

In this study, we explored the association of GGT level and IVIG resistance in KD patients to try to understand the biological mechanism of action underlying these two clinical findings. With our systematic analyses of gene regulation and associated clinical findings and outcomes, we hypothesize: (1) neutrophil apoptosis induced by IVIG plays a pivotal role in IVIG responsiveness; (2) changes in sialylation and GGT levels in acute KD patients may contribute together to IVIG resistance by blocking IVIG-induced neutrophil apoptosis.

## Methods

The normal ranges of GGT and alanine aminotransferase (ALT) are shown in Table A in S1 File and Table B in S1 File respectively, while the normal range of C-reactive protein (CRP) is shown in the footnotes of Table E in S1 File.

### Cohort I: Subjects used to analyze the relationship between the levels of GGT and IVIG resistance

A cohort with 686 subjects with KD (Cohort I) was used to explore the associations between GGT levels and IVIG resistance. All subjects in this cohort were enrolled at Rady Children’s Hospital in San Diego after obtaining written parental informed consent and patient assent as appropriate. All subjects were treated with 2 g/kg of IVIG (Gammagard®) over a 10–12 hour period as per the pharmacy’s standard protocol. The study protocol was conducted in accordance with the Declaration of Helsinki and was approved by Institutional Review Boards of UCSD and Stanford University. KD subjects in this study were: a) patients with fever (≥38.0°C rectally or orally) for no more than 10 days, plus at least four of the five principal clinical criteria, b) patients meeting fewer criteria but with coronary artery abnormalities (CAA) (Z-score ≥2.5 for left anterior descending [LAD] and/or right coronary arteries [RCA]) documented by echocardiogram, and c) patients with fewer than 4 clinical criteria but meeting the American Heart Association (AHA) criteria for incomplete KD by laboratory criteria [4]. KD subjects meeting the inclusion criteria were identified from the database maintained at the UCSD KD Research Center. We obtained prospectively collected demographic and clinical data, the results of laboratory studies prior to IVIG administration, and IVIG-responsiveness. IVIG-resistance was defined as persistent or recurrent fever (rectal or oral temperature ≥38.0°C) at least 36 hours but no longer than 7 days after completion of the initial IVIG infusion (2g/kg) [10]. Subjects in this cohort were either treated with a second course of IVIG or infliximab for IVIG-resistant KD.

### Cohort II: Subjects used for gene expression profiling and GGT/IVIG association analysis

We performed gene expression profiles of whole blood obtained from a sub-cohort of 146 KD subjects (Cohort II) as previously described [16]. Of these KD subjects, 130 had complete data on IVIG responsiveness and serum GGT levels available for subsequent analyses; clinical characteristics of this cohort are summarized in Table C in S1 File. The odds ratios of gene expression stratified by IVIG response were calculated using logistic regression with glm2 in R [17].

For each of the 31,000 genes in the 130 subjects, the explained variations in GGT levels or IVIG responsiveness were estimated using R square of linear regression or the McFadden's pseudo- R square [18, 19] of logistic regression [17], respectively.

**Fig 1:**
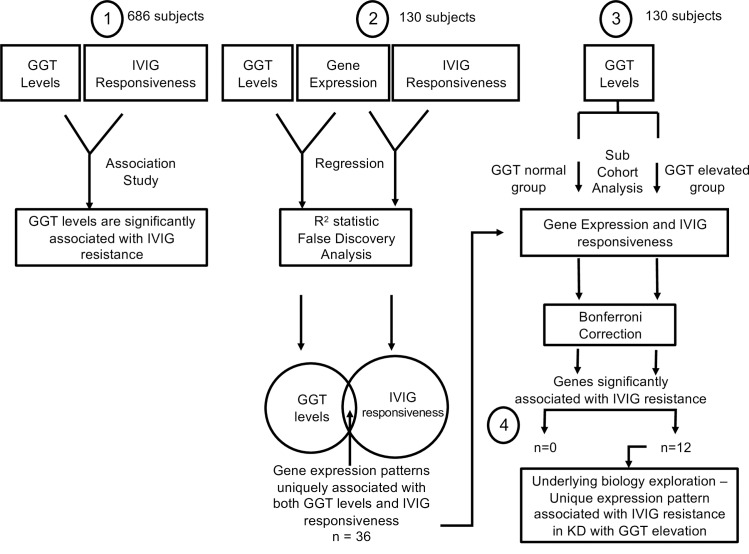
Study outline of analyses to uncover unique gene expression patterns underlying IVIG responsiveness in subjects with elevated serum GGT levels. (1) Analysis of GGT levels with IVIG responsive-ness; (2) Global gene expression analysis by R2 statistic and FDR anal-yses to identify genes, explaining variations in both IVIG responsiveness and GGT elevation; (3) Targeted analysis of the step 2 discovered genes in either GGT normal or elevated subgroups to reveal gene expression patterns specific for each sub-group of subjects; (4) Pathway and literature analysis to explore the underlying biology of IVIG resistance in subjects with GGT elevation.

Correction for multiple hypothesis testing on the explained variations was performed using the permutation-based false discovery rate (FDR) analytical method [20]. FDRs of GGT- and IVIG-associated variations for each gene were estimated using Monte Carlo simulation. Genes with FDRs less than a threshold of 0.01 were considered true discoveries explaining the variations in GGT levels and IVIG responsiveness. The common genes among true discoveries of both GGT and IVIG were used for the next analysis (Fig 1, Panel 2).

The study population was divided into two sub-cohorts, one with normal and the other with elevated GGT levels, using age-specific reference ranges established in our clinical laboratory (Table A in S1 File). Logistic regression was performed for each of the 36 common genes with FDR <1%, to evaluate its contribution to the risk for IVIG resistance in each sub-cohort. The Bonferroni method was applied to correct for multiple hypothesis testing [21, 22].

### Cohorts III and IV: Subjects used to investigate and validate the changes of neutrophils in response to IVIG treatment

Cohort III was derived from the placebo arm of a phase 3, randomized, double-blind, placebo-controlled trial of IVIG with or without infliximab for treatment of KD, which was conducted from March 2009 to August 2012 in two children’s hospitals in the USA [23]. Cohort III included 95 KD patients of the 98 KD patients from the placebo arm, and was used to investigate changes of neutrophils in response to IVIG treatment. The 3 excluded placebo subjects had missing data for IVIG response (n = 1) or absolute neutrophil count (n = 2). The study protocol was reviewed and approved by the Institutional Review Boards at the University of California San Diego’s and Nationwide Children’s Hospital. Written informed consent was obtained from the parents or legal guardians and assent, when appropriate, was obtained from the patient.

To validate our observations on changes of neutrophils in response to IVIG treatment in Cohort III, we utilized another independent cohort (Cohort IV) with 587 KD subjects. Approved by the institutional review board of Korean University Medical Center, the medical records of KD patients from January 2008 to December 2013 were reviewed. The diagnosis and treatment of KD was based on AHA criteria [4]. CAA were diagnosed on the basis of the criteria proposed by the Japanese Kawasaki Disease Research Committee in 1984 [24]. Patients with complete differential leukocyte counts at diagnosis and 2 days after IVIG treatment, IVIG response and CAA data were included. Detailed characteristics, clinical data and results of laboratory studies of subjects were previously described [25].

The absolute neutrophil count (ANC) (mature and immature (band) forms) were calculated for both the UCSD and Korean cohorts.

### General data analysis

All statistical analyses and plots were performed using R 3.2.2 [26] and ggplot2 [27] if not mentioned explicitly. The difference in neutrophil reduction between the IVIG responders and the IVIG non-responders after treatment was tested with the Mann-Whitney U-test. The scatter plot curve of neutrophil reduction versus ANC was done with loess fit.

## Results

### Association between IVIG resistance risk and GGT levels

The 575 IVIG responders and 111 IVIG non-responders in Cohort I had similar demographic and clinical characteristics (Table 1). Subjects were treated with IVIG just after obtaining baseline laboratory values. In addition to elevated GGT levels, IVIG resistant subjects also had significantly higher ALT, CRP values, band percentage, and absolute white blood cell count. This is in line with previous meta-analysis [15] that serum levels of ALT and GGT in IVIG non-responders were significantly higher than that in the IVIG responders.

**Table 1:** Clinical and laboratory characteristics values of 686 IVIG responsive and resistant KD subjects.

	**IVIG responders (N = 575)**	**IVIG non-responders (N = 111)**	***P* value**
Age at diagnosis, years	2.6 (1.4–4.3)	2.4 (1.4–4.2)	NS
Male, N (%)	350 (60.9)	72 (64.9)	NS
Illness day at sample collection days	6 (5–7)	5 (4–6)	NS
Incomplete KD, N (%)	67 (11.7)	9 (8.1)	NS
Coronary artery aneurysms, N (%)	12 (2.1)	5 (4.5)	NS
Ethnicity, N (%)			
Asian	99 (17.2)	14 (12.6)	NS
African-American	23 (4.0)	4 (3.6)	NS
Caucasian	135 (23.5)	30 (27.0)	NS
Hispanic	186 (32.3)	37 (33.3)	NS
More than race	111 (19.3)	24 (21.6)	NS
Other	8 (1.3)	1 (0.9)	NS
CRP, mg/dL	7.0 (4.0–14.6)	8.3 (5.2–18.6)	< 0.01
ESR, mm/h	61 (42–77)	53 (36–68)	< 0.05
WBC, ×10^3^/mm^3^	13.2 (10.6–17.3)	13.6 (10.6–17.4)	NS
ANC, cells/mm^3^	8,836 (6,440–11,696)	9,585 (7,301–12,328)	NS
ZHgb	-1.2 (-2.1–0.42)	-1.2 (-2.2–0)	NS
ALT, IU/L	35 (19–98)	76 (38–142)	< 0.001
GGT, IU/L	37 (17–109)	76 (28–157)	< 0.001
Albumin, g/dL	3.9 (3.5–4.2)	3.7 (3.4–4.0)	NS
Polymorphonuclear leukocytes (%)	54 (42–64)	51.0 (40.0–62.5)	NS
Platelet count, ×10^3^/mm^3^	373 (291–467)	340 (282–430)	NS
Bands (%)	11.0 (4.0–19.0)	18.0 (10.0–32.2)	< 0.001
ABC	1,440 (554–2,634)	2,450 (1,272–4,512)	< 0.001

Values are presented as median (IQR: Interquartile range). KD: Kawasaki disease, IVIG: intravenous immunoglobulin, CRP: C-reactive protein, ESR: erythrocyte sedimentation rate, WBC: white blood count, ANC: absolute neutrophil count, ZHgb: standard deviations from the mean hemoglobin concentration normalized for age, ALT: alanine aminotransferase, GGT: gamma-glutamyl transferase. The difference between groups were tested using Wilcoxon rank sum test, NS: not significant.

**Fig 2:**
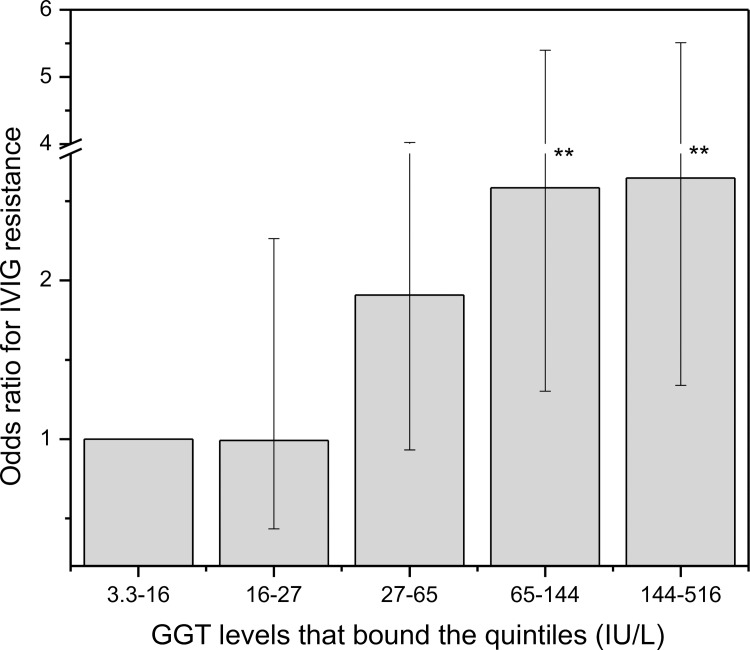
Odds ratio for IVIG resistance per quintile of GGT level. The odds ratios were calculated against the first quintile with the lowest GGT level. The 95% confidential intervals were shown as the error bars. **: *P* value < 0.01 using fisher exact test.

IVIG resistance was examined according to GGT levels quintiles (Fig 2). IVIG resistance risk in each quintile exhibited a progressive association with the GGT levels and the odds ratio reached 2.6 for subjects in the highest quintile compared with the lowest quintile.

### Global gene expression analyses with FDR

**Fig 3:**
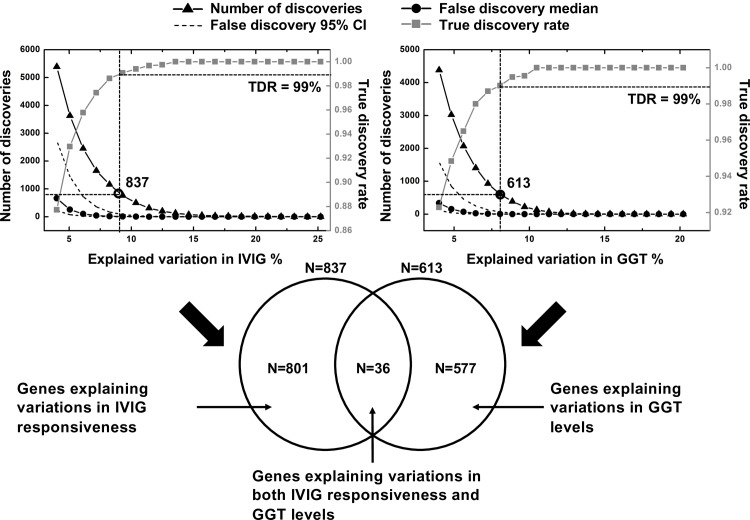
R^2^ statistic and FDR analyses with respect to GGT levels and IVIG responsiveness. Top left panel: discovery of genes explaining variations in IVIG responsiveness. Top right panel: discovery of genes explaining variations in GGT levels. Bottom panel: Venn diagram analysis uncovering genes explaining variations in both IVIG responsiveness and GGT levels.

Of the 130 KD subjects with gene expression data in Cohort II, 100 were IVIG responders and 30 were IVIG non-responders. At the time of diagnosis, 45 subjects had normal serum GGT levels, and 85 had elevated GGT levels (Table C in S1 File). In multiple hypothesis testing of gene expression for GGT levels and IVIG resistance, the number of true discovered genes decreased with increasing R square values (Fig 3). The median of false discoveries decreased much faster than the total number of discoveries because the event, that a gene can explain the significant variations of the randomized response, can only occur by chance. In analyses of IVIG resistance and elevated GGT levels, the R square thresholds corresponding to a TDR of 99% resulted in discovery of 837 and 613 genes, respectively. Of these, 36 were common to both gene sets (Fig 3 bottom panel).

### Candidate gene analysis with normal and elevated GGT level sub-cohorts

In order to explore these 36 genes with regard to the GGT-level-associated IVIG responses, we partitioned Cohort II into sub-cohorts with normal (45 subjects including 38 IVIG responders and 7 non-responders) or elevated GGT levels (85 subjects including 62 IVIG responders and 23 non-responders). After applying the Bonferroni correction, significant associations (*P* value < 0.05) with IVIG resistance persisted for 12 out of the 36 genes among patients with elevated GGT levels and none among patients with normal GGT levels (Table 2). The highest odds ratio was 114.1 for *ST6GALNAC3* in the GGT-elevated subgroup, and exhibited about a 50-fold difference between the subgroups (Table 2).

**Table 2:** List of 12 genes significantly contributing to IVIG resistance in subgroup with elevated GGT levels identified by logistic regression from 36 genes found by R^2^ statistic and FDR analyses.

**Gene symbol**	**HGNC name**	**Normal GGT**		**Elevated GGT**	
		Odds ratio (95% CI)	*P* value^*^	Odds ratio (95% CI)	*P* value^*^
*ST6GALNAC3*	ST6 (alpha-N-acetyl-neuraminyl-2,3-beta-galactosyl-1,3)-N-acetylgalactosaminide alpha-2,6-sialyltransferase 3	2.3 (0.003–2217)		**114.1** (9.2–2540)	0.03
*LAMTOR5*	late endosomal/lysosomal adaptor, MAPK and MTOR activator 5	2.1 (0.3–177.7)		**112.5** (10–2034)	0.02
*CMTM4*	CKLF-like MARVEL transmembrane domain containing 4	0.7 (0.01–23.1)		34.7 (5.5–303.1)	0.02
*LOC653907*	similar to complement component (3b/4b) receptor 1 isoform F precursor (obsolete)	1.2 (0.1–16.5)		19.4 (4.1–119.2)	0.02
*TSHZ3*	teashirt zinc finger homeobox 3	1.1 (0.2–10.4)	> 0.01	14.6 (3.7–72.9)	0.01
*GADD45A*	growth arrest and DNA-damage-inducible, alpha	0.3 (0.04–1.8)		12.2 (4.0–49.4)	0.003
*DACH1*	dachshund family transcription factor 1	0.4 (0.04–3.4)		8.8 (2.9–35.0)	0.02
*PCOLCE2*	procollagen C-endopeptidase enhancer 2	1.2 (0.2–6.4)		4.4 (1.9–11.3)	0.04
*MMP8*	matrix metallopeptidase 8	1.2 (0.2–5.2)		2.8 (1.7–5.2)	0.01
*ATP8B2*	ATPase, aminophospholipid transporter, class I, type 8B, member 2	0.1 (0.005–2.5)		0.1 (0.02–0.4)	0.05
*ABCF1*	ATP-binding cassette, sub-family F (GCN20), member 1	0.2 (0.002–12.0)		0.02 (0.0007–0.2)	0.05
*SSBP3*	single stranded DNA binding protein 3	0.03 (0.0003–1.6)		0.03 (0.0003–0.1)	0.01

* *P* values adjusted by Bonferroni correction.

### Association of the reduced ANC with IVIG treatment outcomes

**Fig 4:**
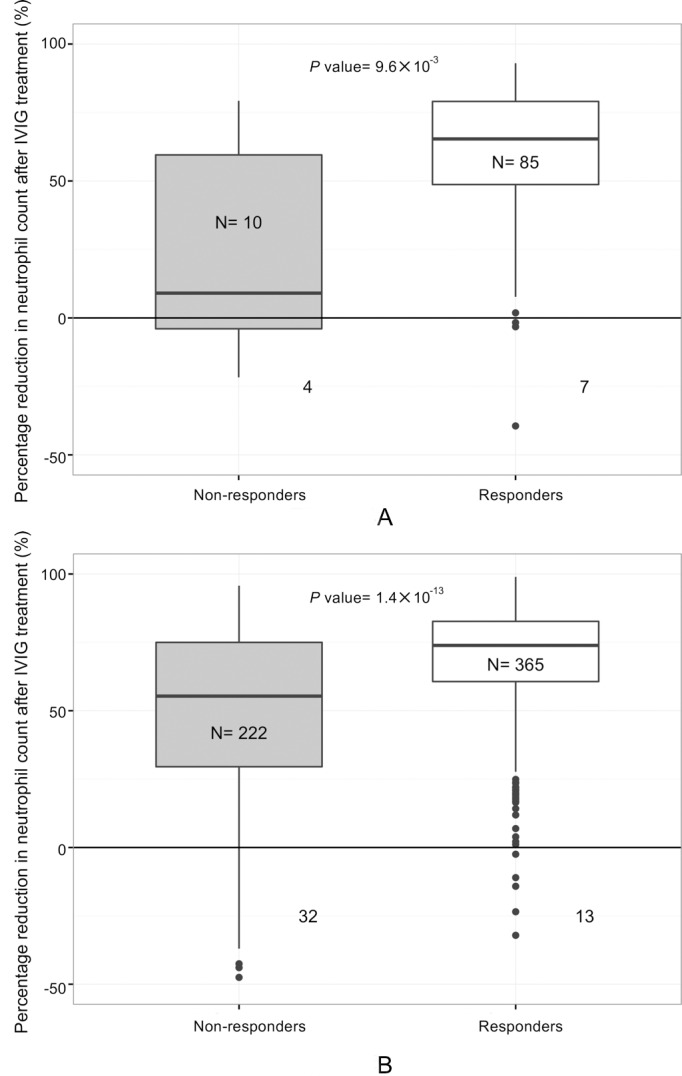
Neutrophil reduction in response to IVIG treatment in two independent cohorts. All *P* values were calculated using Wilcoxon rank sum test. A) Cohort III. B) Cohort IV. The number of patients with rising neutrophil counts after IVIG treatment were indicated by the numbers below x axis alongside each box plot. The *P* values were calculated using Wilcoxon rank sum test.

As shown in Fig 4A, 10 of 95 subjects in Cohort III are IVIG non-responders. Reduction of neutrophil counts after IVIG treatment were common among both IVIG responders (92%) and IVIG non-responders (60%). IVIG non-responders had significantly lower (*P* value 9.6 × 10^−3^) percentage reduction in neutrophil count than responders. A similar pattern was observed in Cohort IV, which included 222 IVIG non-responders and 365 IVIG responders (Fig 4B, *P* value 1.4 × 10^−13^). We further delineated changes in ANC by stratifying patients into quintiles according to the ANC at presentation (S1 Fig). In comparison with non-responders, a significantly greater decline in ANC was observed in responders in every quintile (S1 Fig). The ANC increased in a small fraction of patients in both groups after IVIG treatment: 14.4% of non-responders and 3.5% of responders. We plotted the trending curve of either absolute or percentage of neutrophil reduction against the pretreatment ANC (Cohort III subjects, S2A and S2B Fig; Cohort IV subjects, S2C and S2D Fig). This trending curve showed that neutrophil reduction increased as a function of the pretreatment ANC in both the resistant and responsive subgroups. Thus, Cohort IV analysis validated the Cohort III observations that the IVIG non-responders have less reduction in neutrophil count than the IVIG responders. Within subgroup with elevated GGT levels (S3 Fig), the association between high levels of GGT and neutrophil reduction revealed no clear trend.

## Discussion

Our study highlights the significant association of the elevated GGT levels with IVIG treatment outcomes in KD patients. Global gene expression analysis revealed 36 genes that could explain the variations of both IVIG treatment outcomes and GGT levels in acute KD patients. Of these 36 genes, 12 retained an association with IVIG resistance in the subgroup with elevated GGT levels, while none remained significant in the normal GGT subgroup. In comparison, although both ALT and GGT levels in IVIG resistant patients were significantly higher than that in the IVIG responsive group, none of the 12 GGT-associated genes were significantly associated with IVIG responses in either the normal or elevated ALT subgroups (Table D in S1 File). These observations suggest that a unique gene expression pattern exists in KD subjects with elevated GGT levels, which may account for their higher risk of resistance to IVIG treatment.

**Fig 5:**
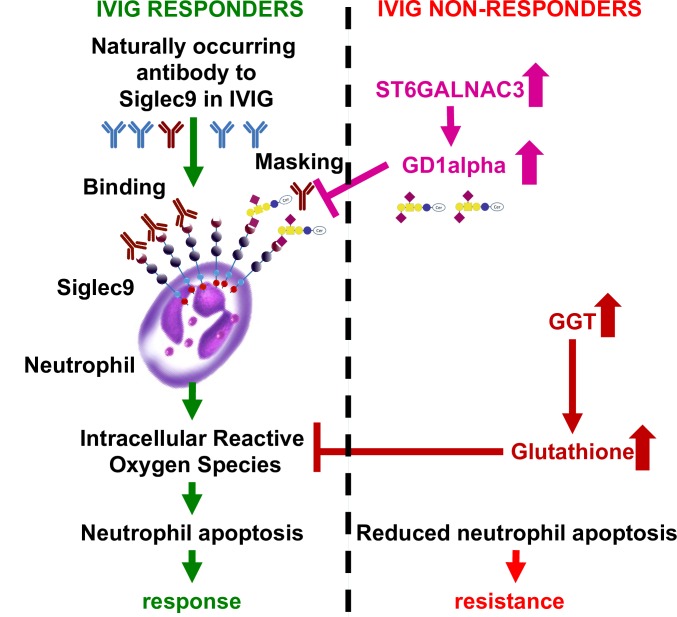
Hypothesis of the underlying biology of IVIG responsiveness involving neutrophils, siglec-9, ST6GALNAC3, and GGT.

Molecular and immunological markers have been shown to segregate and predict responders and non-responders to IVIG therapy [28]. Consistent with previous observations of significantly elevated neutrophil counts in IVIG-nonresponsive patients, the circulating levels of inflammatory mediators including granulocyte-colony stimulating factor (G-CSF) were significantly higher in IVIG non-responders [29] and were positively correlated with higher levels of matrix metalloproteinase-8 (MMP-8), which is 1 of the 12 GGT associated genes found in this study [29, 30]. We reasoned that the 12 GGT associated differentially expressed genes were novel, and may provide molecular insight on the IVIG response. We focused the analyses on *ST6GALNAC3*, which was found to have the highest odds ratio in the elevated GGT group and about a 50-fold greater risk compared to the normal GGT group. *ST6GALNAC3* is a sialyltransferase that exclusively utilizes α2, 3-sialylated ganglioside GM1b as a donor to synthesize ganglioside GD1α by adding a α2, 6-sialic acid onto β-galactoside [31]. The branching α2, 6-sialic acid, could potentially increase the binding affinity of GD1α to siglec-9, a sialic acid-binding immunoglobulin-type lectin which preferably binds to α 2,3- and α 2,6-sialyl residues [32] on monocytes and neutrophils [33] (Fig 5). We observed that the ANC declined in response to IVIG in both IVIG responders and non-responders (Fig 4B). Given that IVIG non-responders are still febrile with ongoing inflammation, we hypothesized a direct impact of IVIG on neutrophils instead of a secondary therapeutic effect of global inflammation reduction. This is consistent with previous smaller cohort observations [34, 35]. Neutrophils isolated from IVIG responders exhibit accelerated spontaneous apoptosis *in vitro* [36], suggesting that apoptosis may cause the reduction of neutrophils during IVIG treatment. Conversely, neutrophils isolated from IVIG non-responders were less inclined to undergo apoptosis in vitro [36]. These observations are in line with our hypothesis that neutrophils in non-responders may be more resistant to IVIG induced apoptosis. In addition, neutrophil count was used as one of the clinical features for IVIG resistance score [37]. Circulating neutrophils and their over activation maybe one of the underlying progressive indicators in IVIG resistance and the severity of the heart lesion [38, 39].

Despite the fact there have been reports of KD occurring in the presence of Autoimmune neutropenia (AIN)[40, 41], for the majority of KD patients, the neutrophil count is elevated. We showed that IVIG non-responders had significantly lower percentage reduction in neutrophil count than responders. The observation of KD symptoms[41] with neutropenia suggested that there might be two KD subgroups with different underlying mechanisms of pathophysiology involving neutrophils. We hypothesize that, within a small subgroup of KD, neutrophil may not necessarily be involved in the pathogenesis and manifestation of KD but rather in the progression and severity of coronary artery lesions (CAL). One explanation in the AIN case with KD is that the neutrophil count sampled in peripheral blood does not always reflect the neutrophil count in the marginated pool or in the tissues. Autopsy data clearly show that neutrophils are the “first responders” in the tissues. Before they can enter the arterial wall, they must marginate. Neither the marginated pool nor the tissue infiltrating neutrophils are measured when blood is drawn. Therefore, it is possible, in the minor subgroup of AIN with KD, neutrophils may still be central to the pathophysiology of the disease even though the numbers measured in the flowing portion of the peripheral blood is low.

Naturally occurring antibodies (Nabs) against siglec-9 (Nabs-siglec-9) are found in IVIG and can induce apoptosis in neutrophils [42–44]. Moreover, this apoptosis is accelerated by proinflammatory cytokines. Granulocyte/macrophage colony-stimulating factor (GM-CSF) and interferon-γ (IFN- γ), which are often upregulated in KD [45, 46]. Therefore, we postulate that Nabs-siglec-9 in IVIG can induce neutrophil apoptosis in KD, explaining the association between the neutrophil reduction and therapeutic outcomes of IVIG (Fig 5): (1) the high expression level of *ST6GALNAC3* leads to increased enzymatic activity of *ST6GALNAC3*, producing more GD1α, which in turn binds to siglec-9 and prevents its recognition by Nabs-siglec-9 in IVIG; (2) elevation of GGT levels will increase the degradation of extracellular glutathione (GSH) to provide cysteine for *de novo* synthesis of intracellular GSH, reducing the intracellular reactive oxygen species (ROS) in neutrophils. Reduction of ROS in neutrophils decreases neutrophil apoptosis induced by IVIG in the presence of GM-CSF and IFN- γ [44]. However, IVIG works through a myriad of paths. Neutrophil apoptosis is probably one of MANY mechanisms by which IVIG may reduce inflammation.

## Conclusions

To the best of our knowledge, we are the first to integrate multifaceted data sets of expression profiles, clinical parameters and outcomes to explore KD pathophysiology. We demonstrated that KD subjects with elevated GGT levels have a unique gene expression pattern that overlaps with the gene expression pattern associated with IVIG resistance. Our study suggests that reduction of circulating neutrophils is one of the hallmarks of the therapeutic effects of IVIG.

Neutrophil activation and intracellular ROS effect due to elevated GGT levels may not be directly associated with KD susceptibility but be mechanistically critical in IVIG resistance and heart lesion pathogenesis [38, 39, 47]. Therefore, both increased *ST6GALNAC3* and elevated GGT levels may lead to reduced neutrophil apoptosis, and consequently IVIG resistance (Fig 5).

We propose two future testable hypotheses: (1) Nabs against siglec-9 (Nabs-siglec-9) in IVIG can induce apoptosis in neutrophils and contribute to the efficacy of IVIG in the treatment of KD. (2) Increased sialylation of gangliosides block IVIG-mediated neutrophil apoptosis and lead to persistent inflammation and IVIG resistance.

If confirmed, our findings may account for the variable effectiveness of different IVIG lot preparations [48, 49], potentially allowing a new quality control approach. Monoclonal antibodies against Siglec-3 and Siglec-2 are in clinical trials [50]. If developed, therapies to induce neutrophil apoptosis could be a more effective KD treatment.

## Supporting Information

S1 Fig Neutrophil reduction in response to IVIG treatment in different quintiles of ANC before treatment in Cohort IV. Numbers below the x axis are numbers of subjects whose ANC increased after IVIG treatment. Of the 13 IVIG responders with ANC increased after IVIG, 12 fell into the lowest quintile of acute phase neutrophils, and none in the last three quintiles.
(TIF)

S2 Fig **Loess curve analyses of neutrophil reduction or the percentage reduction in neutrophil count as a function of the pretreatment ANC (A-B: Cohort III; C-D: Cohort IV).**
(TIFF)

S3 Fig The percentage reduction in neutrophil count as a function of the IVIG GGT levels in subjects with elevated GGT serum levels. (TIF)

S1 File **Table A in S1 File. The standard range for normal GGT (IU/L); Table B in S1 File. The standard range for normal ALT (IU/L); Table C in S1 File.** The clinical characteristics and laboratory values for subjects selected for gene expression analysis; Table D in S1 File. Association of 12 genes with IVIG response in subgroup with normal and elevated CRP levels; Table E in S1 File. Association of 12 genes with IVIG response in subgroup with normal and elevated CRP levels.
(PPTX)
