# Supplementary figures and images for: Unique Molecular Patterns Uncovered in Kawasaki Disease Patients with Elevated Serum Gamma Glutamyl Transferase Levels: Implications for Intravenous Immunoglobulin Responsiveness
Source: PLoS One. 2016 Dec 21;11(12):e0167434. doi: 10.1371/journal.pone.0167434 (PMC5176264; doi:10.1371/journal.pone.0167434)

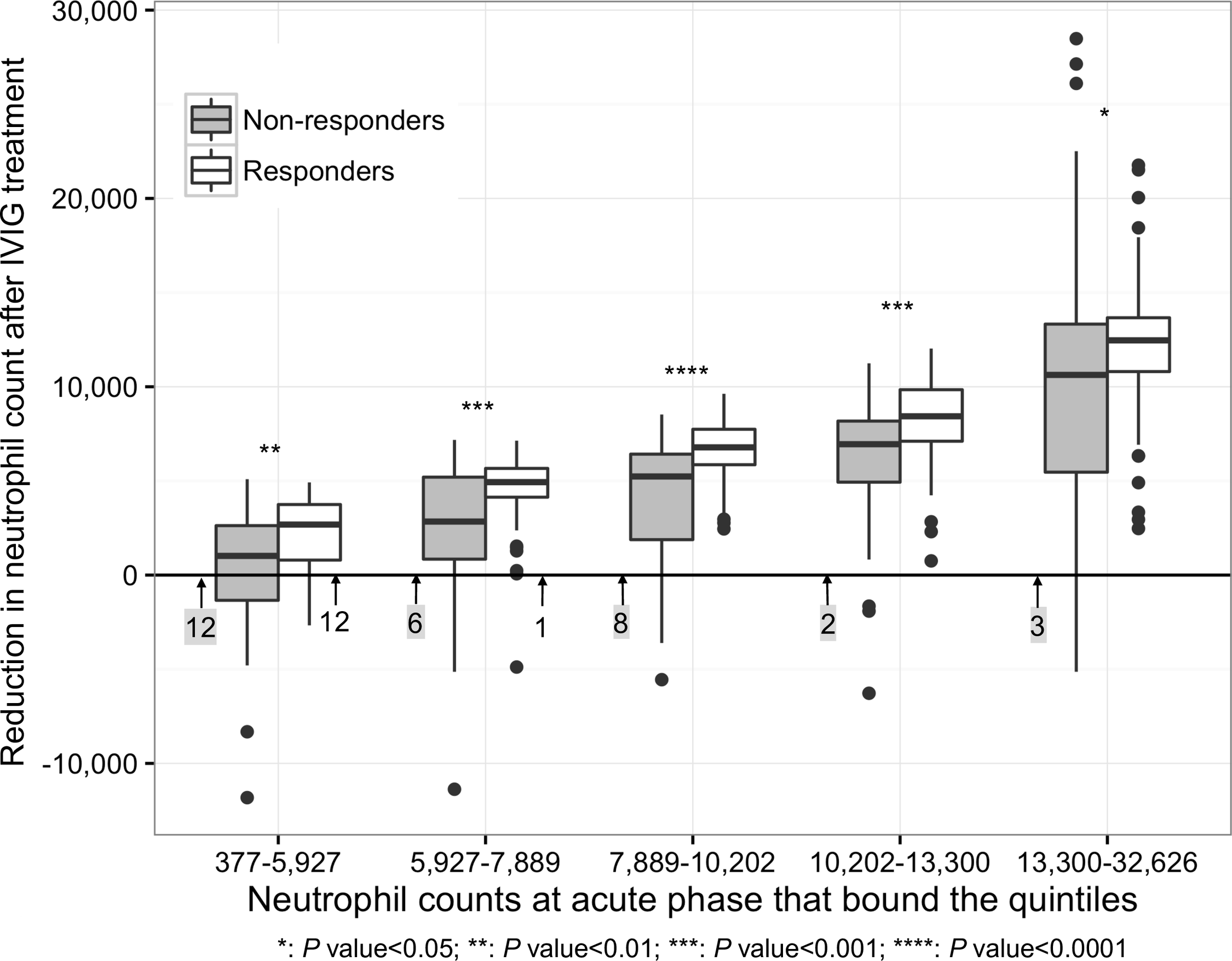

Supplement: S1 Fig — Numbers below the x axis are numbers of subjects whose ANC increased after IVIG treatment. Of the 13 IVIG responders with ANC increased after IVIG, 12 fell into the lowest quintile of acute phase neutrophils, and none in the last three quintiles. (TIF) [file pone.0167434.s001.tif]

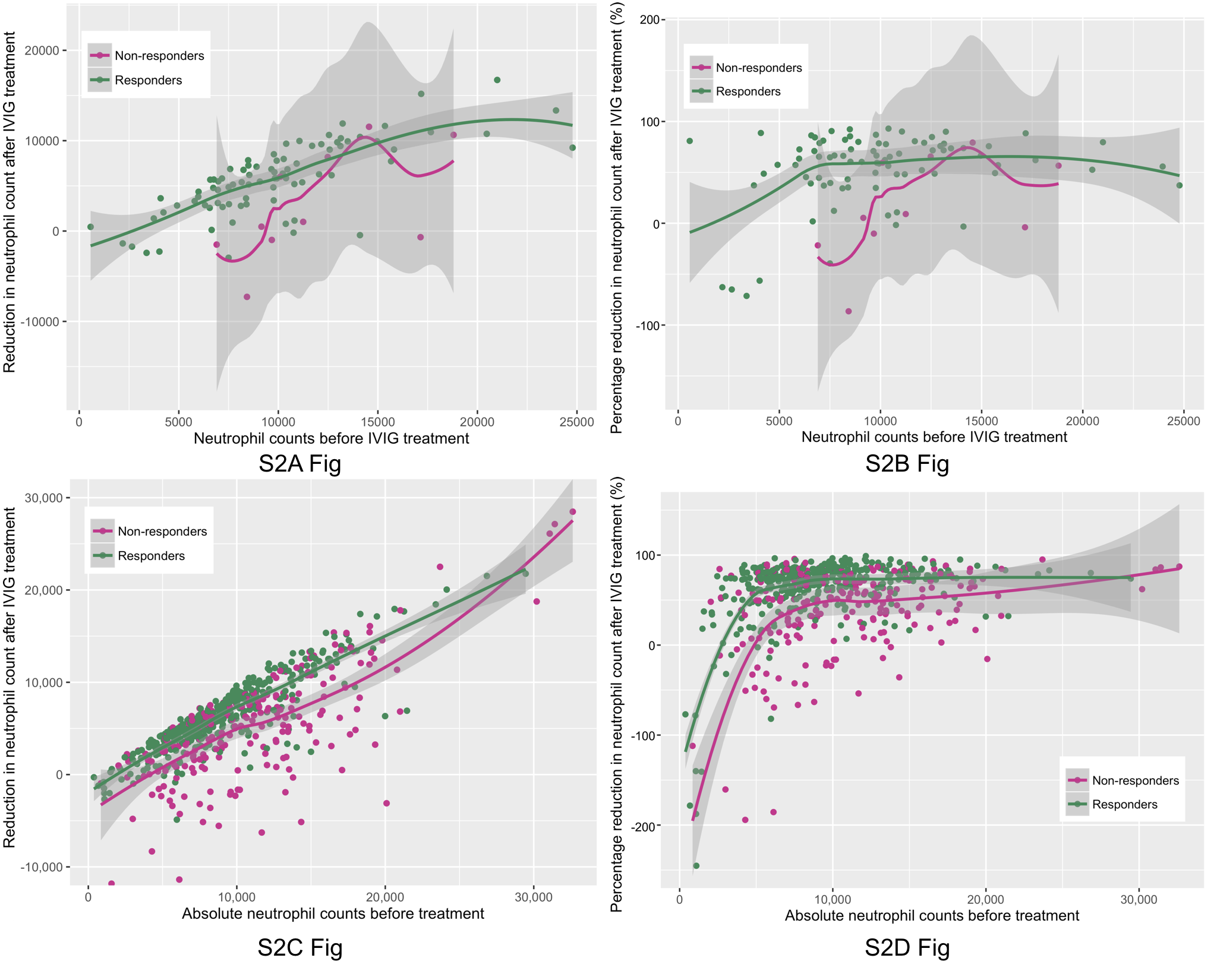

Supplement: S2 Fig — Loess curve analyses of neutrophil reduction or the percentage reduction in neutrophil count as a function of the pretreatment ANC (A-B: Cohort III; C-D: Cohort IV). (TIFF) [file pone.0167434.s002.tiff]

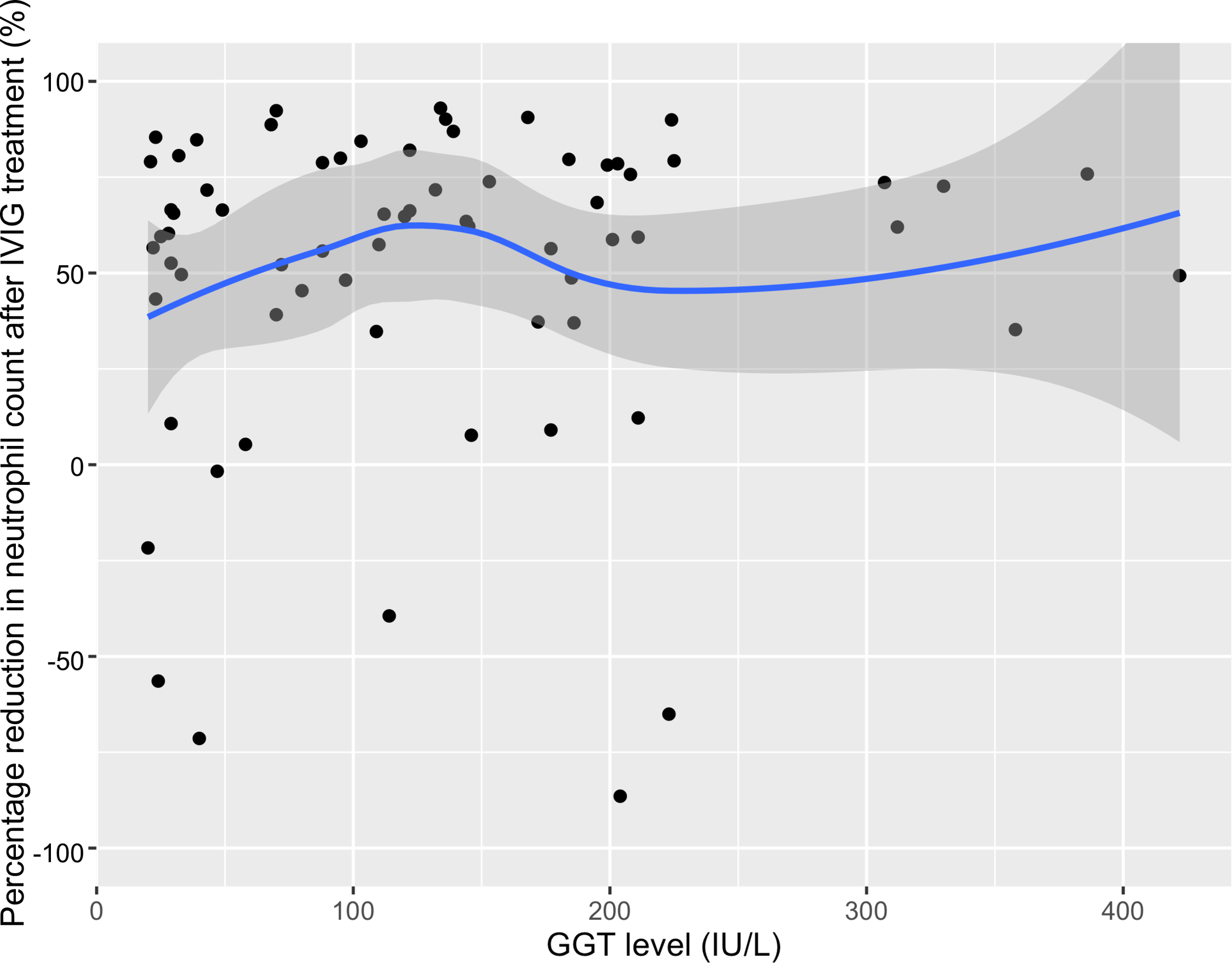

Supplement: S3 Fig — (TIF) [file pone.0167434.s003.tif]
