# Supplementary material for: Unique Molecular Patterns Uncovered in Kawasaki Disease Patients with Elevated Serum Gamma Glutamyl Transferase Levels: Implications for Intravenous Immunoglobulin Responsiveness
Source: PLoS One. 2016 Dec 21;11(12):e0167434. doi: 10.1371/journal.pone.0167434 (PMC5176264; doi:10.1371/journal.pone.0167434)
Supplement: S1 File — Table A in S1 File. The standard range for normal GGT (IU/L); Table B in S1 File. The standard range for normal ALT (IU/L); Table C in S1 File. The clinical characteristics and laboratory values for subjects selected for gene expression analysis; Table D in S1 File. Association of 12 genes with IVIG response in subgroup with normal and elevated CRP levels; Table E in S1 File. Association of 12 genes with IVIG response in subgroup with normal and elevated CRP levels. (PPTX) [file pone.0167434.s004.pptx]

## Slide 1
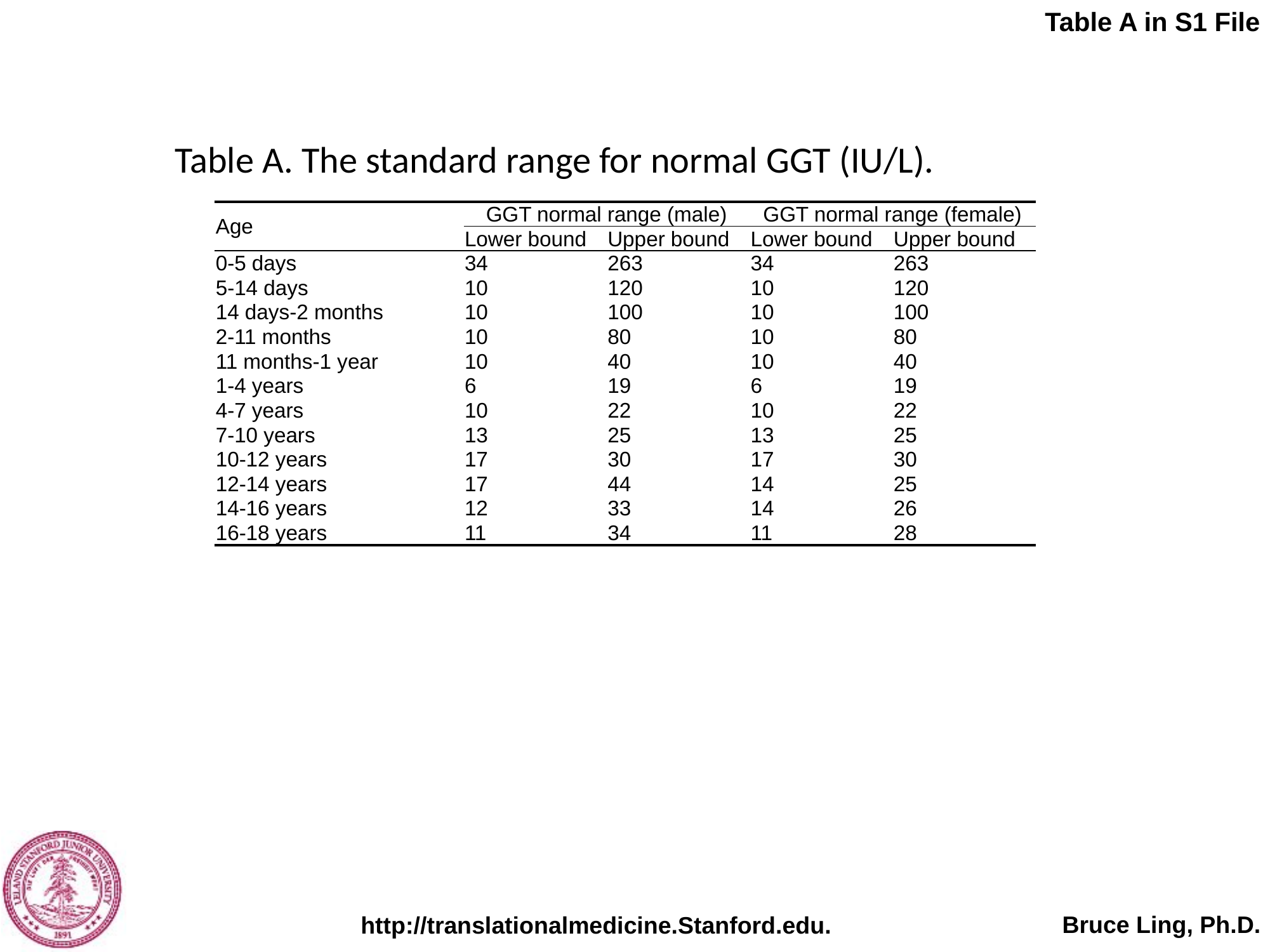

Table A in S1 File
# Table A. The standard range for normal GGT (IU/L).
| Age | GGT normal range (male) | | GGT normal range (female) | |
| --- | --- | --- | --- | --- |
| | Lower bound | Upper bound | Lower bound | Upper bound |
| 0-5 days | 34 | 263 | 34 | 263 |
| 5-14 days | 10 | 120 | 10 | 120 |
| 14 days-2 months | 10 | 100 | 10 | 100 |
| 2-11 months | 10 | 80 | 10 | 80 |
| 11 months-1 year | 10 | 40 | 10 | 40 |
| 1-4 years | 6 | 19 | 6 | 19 |
| 4-7 years | 10 | 22 | 10 | 22 |
| 7-10 years | 13 | 25 | 13 | 25 |
| 10-12 years | 17 | 30 | 17 | 30 |
| 12-14 years | 17 | 44 | 14 | 25 |
| 14-16 years | 12 | 33 | 14 | 26 |
| 16-18 years | 11 | 34 | 11 | 28 |

## Slide 2
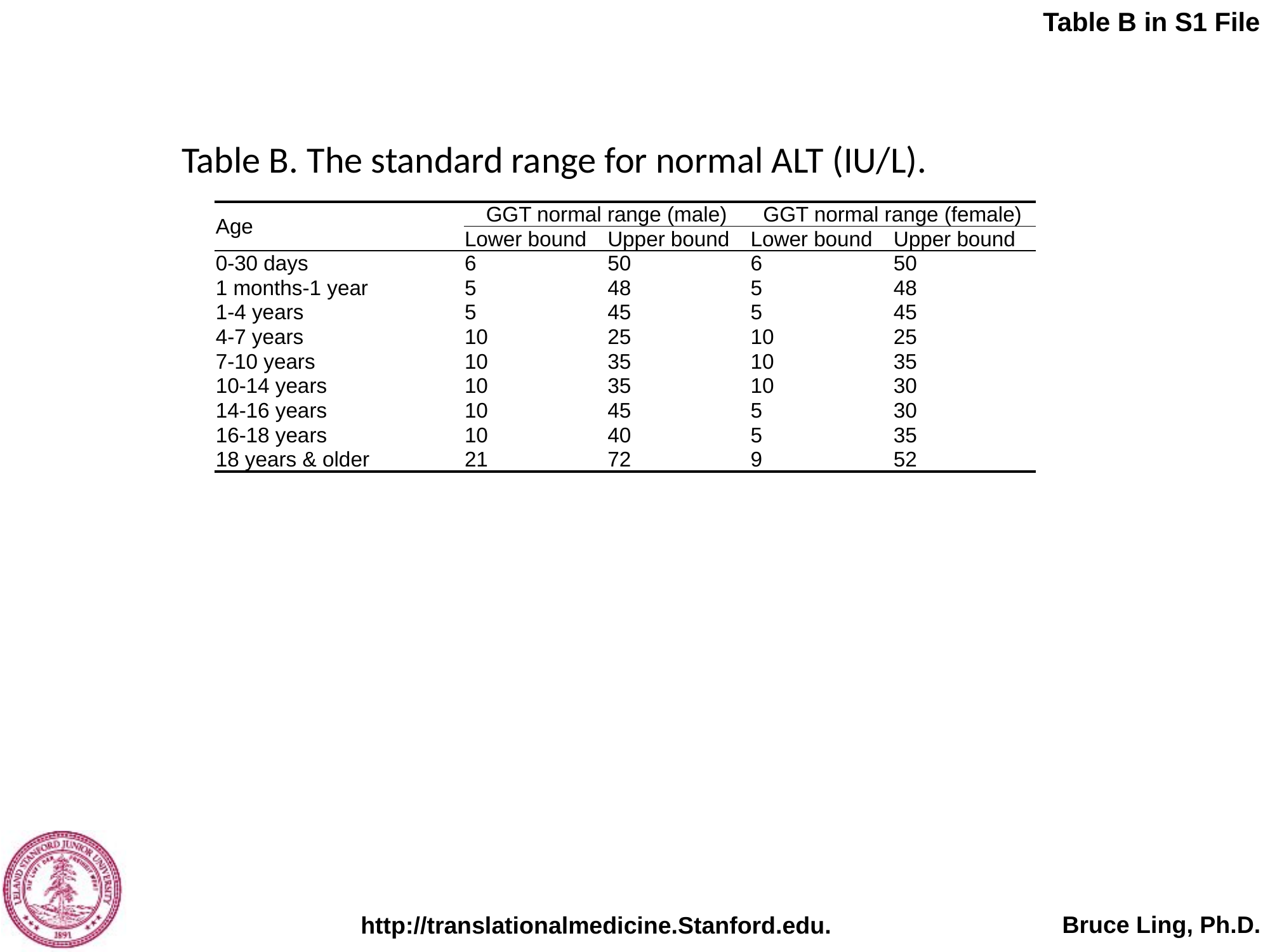

Table B in S1 File
# Table B. The standard range for normal ALT (IU/L).
| Age | GGT normal range (male) | | GGT normal range (female) | |
| --- | --- | --- | --- | --- |
| | Lower bound | Upper bound | Lower bound | Upper bound |
| 0-30 days | 6 | 50 | 6 | 50 |
| 1 months-1 year | 5 | 48 | 5 | 48 |
| 1-4 years | 5 | 45 | 5 | 45 |
| 4-7 years | 10 | 25 | 10 | 25 |
| 7-10 years | 10 | 35 | 10 | 35 |
| 10-14 years | 10 | 35 | 10 | 30 |
| 14-16 years | 10 | 45 | 5 | 30 |
| 16-18 years | 10 | 40 | 5 | 35 |
| 18 years & older | 21 | 72 | 9 | 52 |

## Slide 3
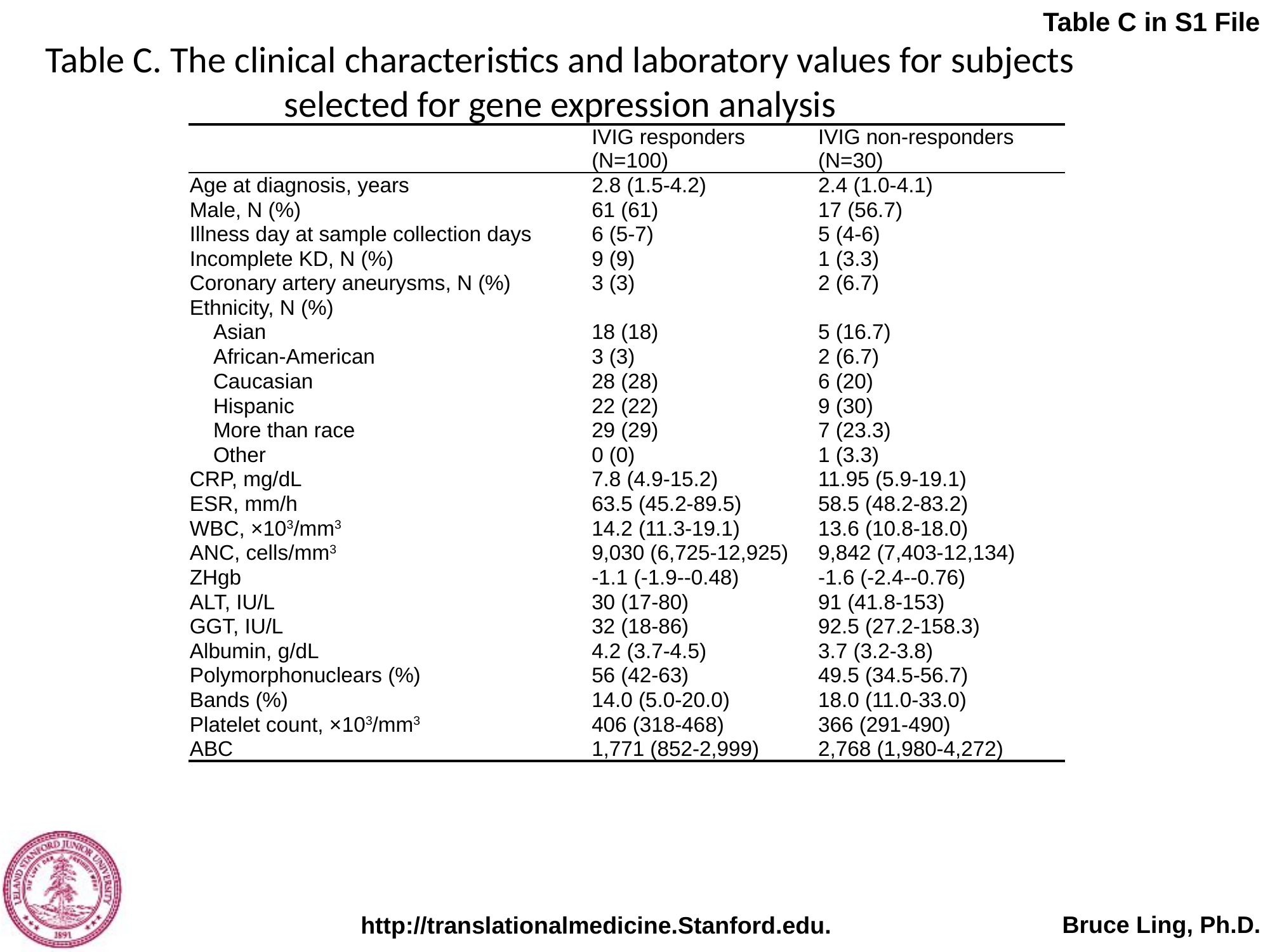

Table C in S1 File
# Table C. The clinical characteristics and laboratory values for subjects selected for gene expression analysis
| | IVIG responders (N=100) | IVIG non-responders (N=30) |
| --- | --- | --- |
| Age at diagnosis, years | 2.8 (1.5-4.2) | 2.4 (1.0-4.1) |
| Male, N (%) | 61 (61) | 17 (56.7) |
| Illness day at sample collection days | 6 (5-7) | 5 (4-6) |
| Incomplete KD, N (%) | 9 (9) | 1 (3.3) |
| Coronary artery aneurysms, N (%) | 3 (3) | 2 (6.7) |
| Ethnicity, N (%) | | |
| Asian | 18 (18) | 5 (16.7) |
| African-American | 3 (3) | 2 (6.7) |
| Caucasian | 28 (28) | 6 (20) |
| Hispanic | 22 (22) | 9 (30) |
| More than race | 29 (29) | 7 (23.3) |
| Other | 0 (0) | 1 (3.3) |
| CRP, mg/dL | 7.8 (4.9-15.2) | 11.95 (5.9-19.1) |
| ESR, mm/h | 63.5 (45.2-89.5) | 58.5 (48.2-83.2) |
| WBC, ×103/mm3 | 14.2 (11.3-19.1) | 13.6 (10.8-18.0) |
| ANC, cells/mm3 | 9,030 (6,725-12,925) | 9,842 (7,403-12,134) |
| ZHgb | -1.1 (-1.9--0.48) | -1.6 (-2.4--0.76) |
| ALT, IU/L | 30 (17-80) | 91 (41.8-153) |
| GGT, IU/L | 32 (18-86) | 92.5 (27.2-158.3) |
| Albumin, g/dL | 4.2 (3.7-4.5) | 3.7 (3.2-3.8) |
| Polymorphonuclears (%) | 56 (42-63) | 49.5 (34.5-56.7) |
| Bands (%) | 14.0 (5.0-20.0) | 18.0 (11.0-33.0) |
| Platelet count, ×103/mm3 | 406 (318-468) | 366 (291-490) |
| ABC | 1,771 (852-2,999) | 2,768 (1,980-4,272) |

## Slide 4
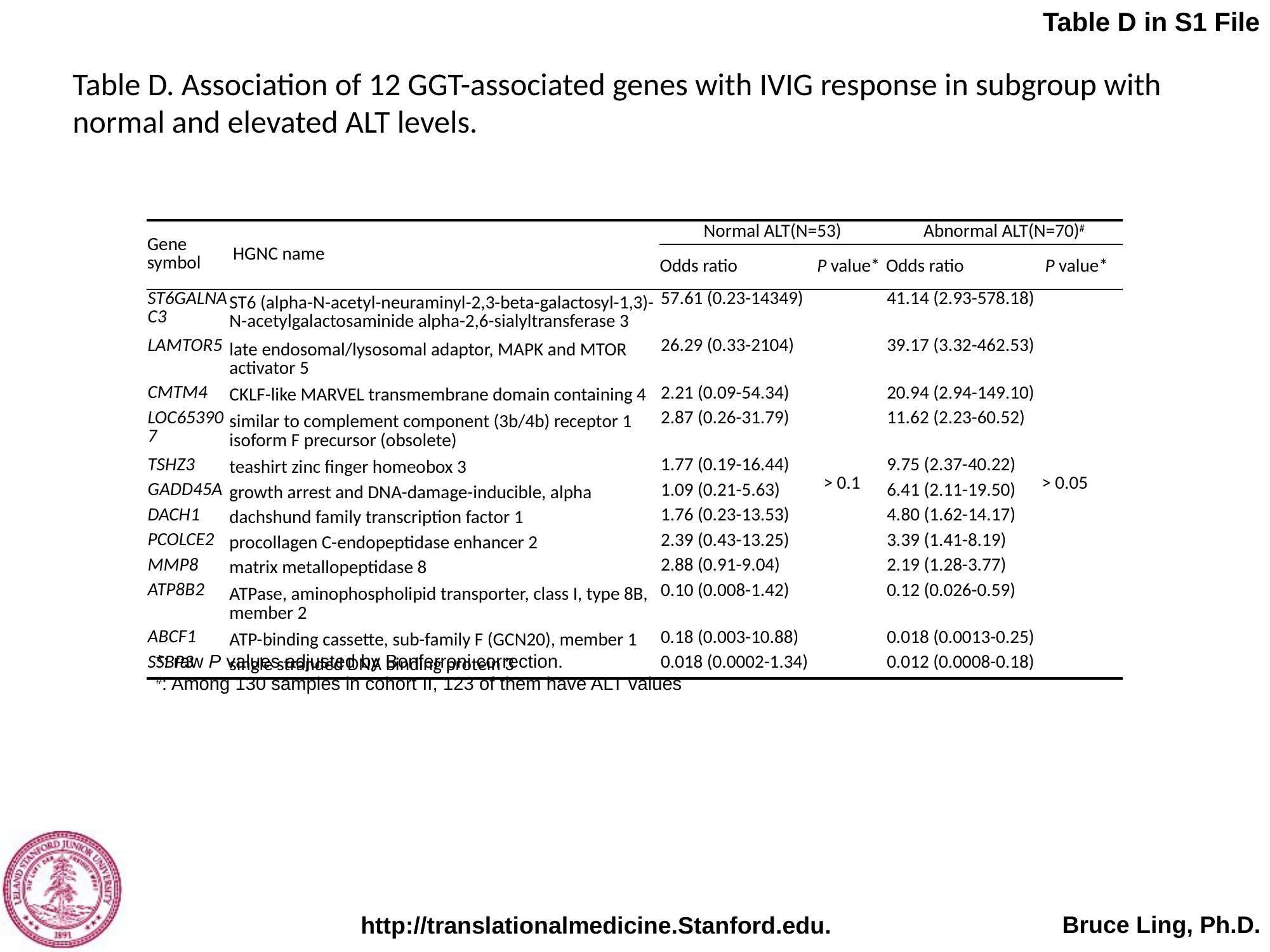

Table D in S1 File
# Table D. Association of 12 GGT-associated genes with IVIG response in subgroup with normal and elevated ALT levels.
| Gene symbol | HGNC name | Normal ALT(N=53) | | Abnormal ALT(N=70)# | |
| --- | --- | --- | --- | --- | --- |
| | | Odds ratio | P value\* | Odds ratio | P value\* |
| ST6GALNAC3 | ST6 (alpha-N-acetyl-neuraminyl-2,3-beta-galactosyl-1,3)-N-acetylgalactosaminide alpha-2,6-sialyltransferase 3 | 57.61 (0.23-14349) | > 0.1 | 41.14 (2.93-578.18) | > 0.05 |
| LAMTOR5 | late endosomal/lysosomal adaptor, MAPK and MTOR activator 5 | 26.29 (0.33-2104) | | 39.17 (3.32-462.53) | |
| CMTM4 | CKLF-like MARVEL transmembrane domain containing 4 | 2.21 (0.09-54.34) | | 20.94 (2.94-149.10) | |
| LOC653907 | similar to complement component (3b/4b) receptor 1 isoform F precursor (obsolete) | 2.87 (0.26-31.79) | | 11.62 (2.23-60.52) | |
| TSHZ3 | teashirt zinc finger homeobox 3 | 1.77 (0.19-16.44) | | 9.75 (2.37-40.22) | |
| GADD45A | growth arrest and DNA-damage-inducible, alpha | 1.09 (0.21-5.63) | | 6.41 (2.11-19.50) | |
| DACH1 | dachshund family transcription factor 1 | 1.76 (0.23-13.53) | | 4.80 (1.62-14.17) | |
| PCOLCE2 | procollagen C-endopeptidase enhancer 2 | 2.39 (0.43-13.25) | | 3.39 (1.41-8.19) | |
| MMP8 | matrix metallopeptidase 8 | 2.88 (0.91-9.04) | | 2.19 (1.28-3.77) | |
| ATP8B2 | ATPase, aminophospholipid transporter, class I, type 8B, member 2 | 0.10 (0.008-1.42) | | 0.12 (0.026-0.59) | |
| ABCF1 | ATP-binding cassette, sub-family F (GCN20), member 1 | 0.18 (0.003-10.88) | | 0.018 (0.0013-0.25) | |
| SSBP3 | single stranded DNA binding protein 3 | 0.018 (0.0002-1.34) | | 0.012 (0.0008-0.18) | |
*: raw P values adjusted by Bonferroni correction.
#: Among 130 samples in cohort II, 123 of them have ALT values

## Slide 5
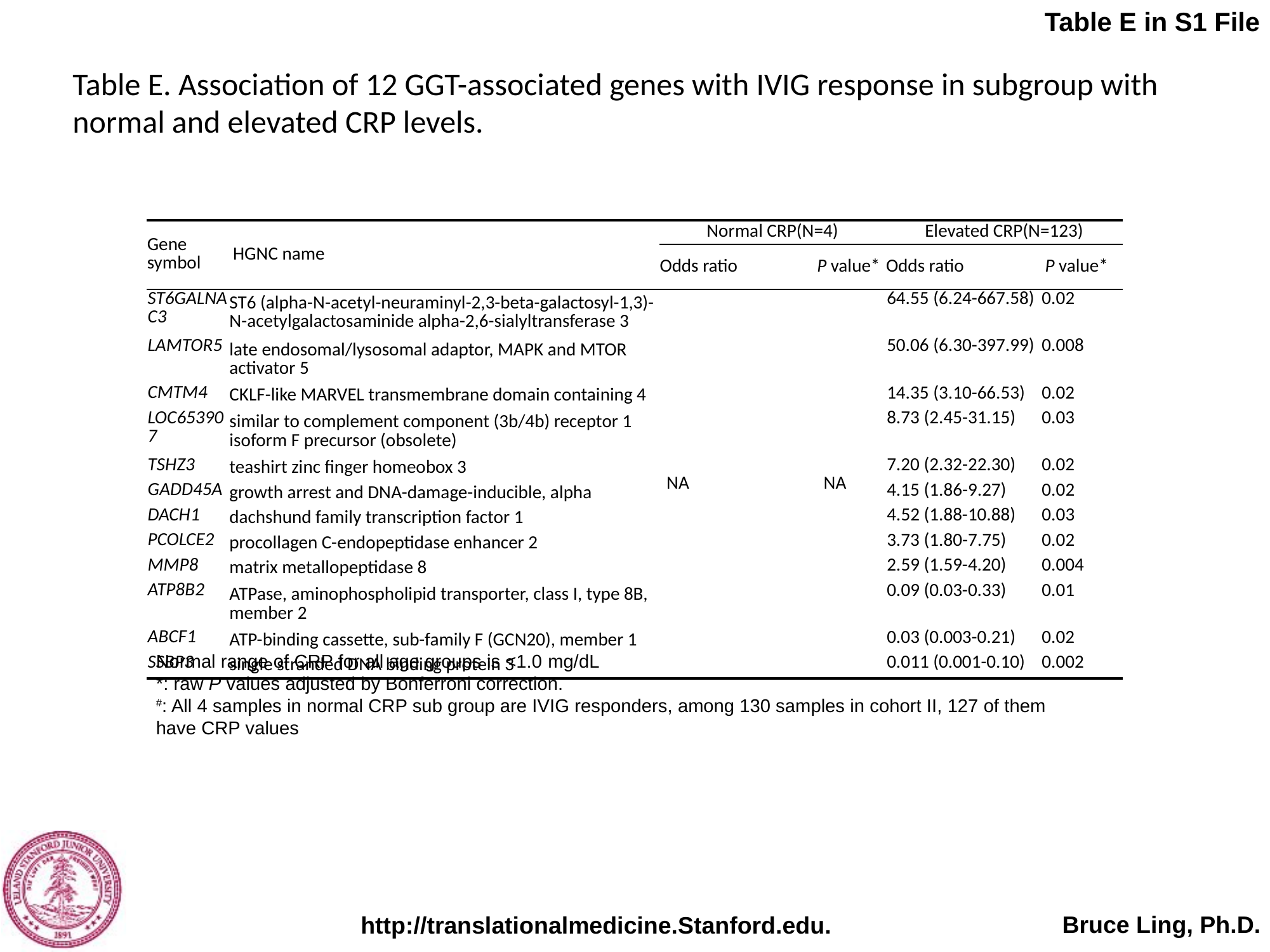

Table E in S1 File
# Table E. Association of 12 GGT-associated genes with IVIG response in subgroup with normal and elevated CRP levels.
| Gene symbol | HGNC name | Normal CRP(N=4) | | Elevated CRP(N=123) | |
| --- | --- | --- | --- | --- | --- |
| | | Odds ratio | P value\* | Odds ratio | P value\* |
| ST6GALNAC3 | ST6 (alpha-N-acetyl-neuraminyl-2,3-beta-galactosyl-1,3)-N-acetylgalactosaminide alpha-2,6-sialyltransferase 3 | NA | NA | 64.55 (6.24-667.58) | 0.02 |
| LAMTOR5 | late endosomal/lysosomal adaptor, MAPK and MTOR activator 5 | | | 50.06 (6.30-397.99) | 0.008 |
| CMTM4 | CKLF-like MARVEL transmembrane domain containing 4 | | | 14.35 (3.10-66.53) | 0.02 |
| LOC653907 | similar to complement component (3b/4b) receptor 1 isoform F precursor (obsolete) | | | 8.73 (2.45-31.15) | 0.03 |
| TSHZ3 | teashirt zinc finger homeobox 3 | | | 7.20 (2.32-22.30) | 0.02 |
| GADD45A | growth arrest and DNA-damage-inducible, alpha | | | 4.15 (1.86-9.27) | 0.02 |
| DACH1 | dachshund family transcription factor 1 | | | 4.52 (1.88-10.88) | 0.03 |
| PCOLCE2 | procollagen C-endopeptidase enhancer 2 | | | 3.73 (1.80-7.75) | 0.02 |
| MMP8 | matrix metallopeptidase 8 | | | 2.59 (1.59-4.20) | 0.004 |
| ATP8B2 | ATPase, aminophospholipid transporter, class I, type 8B, member 2 | | | 0.09 (0.03-0.33) | 0.01 |
| ABCF1 | ATP-binding cassette, sub-family F (GCN20), member 1 | | | 0.03 (0.003-0.21) | 0.02 |
| SSBP3 | single stranded DNA binding protein 3 | | | 0.011 (0.001-0.10) | 0.002 |
Normal range of CRP for all age groups is <1.0 mg/dL
*: raw P values adjusted by Bonferroni correction.
#: All 4 samples in normal CRP sub group are IVIG responders, among 130 samples in cohort II, 127 of them have CRP values
